# Modern Mathematics in Biomedical Imaging

**DOI:** 10.1155/2011/618972

**Published:** 2011-04-28

**Authors:** Yangbo Ye, Robert J. Plemmons, James G. Nagy, Qinian Jin

**Affiliations:** ^1^Department of Mathematics, The University of Iowa, Iowa City, IA 52242, USA; ^2^Department of Computer Science and Department of Mathematics, Wake Forest University, Winston-Salem, NC 27109, USA; ^3^Department of Mathematics and Computer Science, Emory University, Atlanta, GA 30322, USA; ^4^Department of Mathematics, Virginia Tech, Blacksburg, VA 24061, USA

Over the past decades, modern mathematics is playing an increasingly important role in the development of biomedical imaging, yielding groundbreaking ideas and powerful tools for clinical and preclinical applications. Nevertheless, there are not only growing obstacles between applied mathematicians and imaging engineers to communicate more effectively but also great synergy to be unlocked across boundaries between mathematics and imaging technology. Suggested by the Editor-in-Chief, we organized this special issue to address theseunique challenges by presenting some sophisticated mathematical methods in an engineer-friendly language and demonstrating some representative applications of contemporary analytic tools in image reconstruction research.

Nonrigid image registration and a diffeomorphic version of the demons algorithm have been commonly used to estimate tissue deformations in highly deformable anatomies. In the paper by G. Janssens et al. in this issue, the authors further developed the concept of continuous diffeomorphic flow and proposed a diffeomorphic version of the morphons registration method. This method can be used to obtain accurate estimation of deformations between images with variable contrast and hence can be applied to radiotherapy for lung cancer patients and to 4D respiratory-correlated CT of the thorax. 

Bioluminescence tomography (BLT) has been developed at a fast pace in recent years in study of physiological and pathological processes at cellular and molecular levels. In practice, fine discretization is required but may cause large datasets and an increase the ill-posedness of the problem. In the paper by J. Liu et al. in this issue, the authors present a multilevel sparse reconstruction method based on a framework of the finite-element method. Empirical results showed its effectiveness and potential applications in BLT. 

When one considers the time-integrated X-ray flux from multiple X-ray sources to shorten the data acquisition process, a promising way is to use overlapped projections from multiple X-ray sources. In order to perform image reconstruction effectively and efficiently from overlapping projections in this configuration, H. Yu et al. developed a multisource simultaneous-algebraic reconstruction technique regularized by a sparsity-oriented constraint in the soft-threshold filtering framework in their paper in this issue. Their numerical simulation further verified the proposed algorithm and demonstrated its advantages in image reconstruction from overlapping data.

Mathematical modeling in dynamic positron emission tomography (PET) can be simplified, using compartment models as a linear system. To avoid invasive arterial sampling of blood to acquire values of tracer concentration, blind methods to estimate both blood input and kinetic parameters have recently drawn attention. In a paper by Y. Cheng and I. Ş. Yetik in this issue, the authors presented a method to bound errors in kinetic parameter estimates. Their findings are important to show the effect of the blood function on kinetic parameter estimation and to evaluate various blind methods.

In 3D parallel-beam geometry of computed tomography, Y. Wei et al. demonstrated in a paper in this issue that the inverse Fourier transform in different coordinate systems leads to different reconstruction formulas. They explained the reason why the Radon formula cannot be applied to truncated projections. They then introduced a Γ coordinate system, computed its weight functions for the inverse Fourier transform, and obtained a simplified scanning model. Their results on the motion of frequency can be related to the classical Frenet-Serret theorem on 3D curves.

In a paper by A. Becciu et al. in this issue, the authors developed a new formulation to detect critical points of 3D scalar images. Their approach was based on a topological number, which is the 3D generalization to three of the 2D winding numbers. They applied their new method to detection and counting of ovarian follicles and neuronal cells and to the estimation of cardiac motion from tagged MR images. 

The blood vessels and nerve trees consist of tubular objects interconnected into a complex, tree or web-like, geometric structure. Their large-scale range presents a measurement difficulty and an exponential increase of component numbers with decreasing scale. O. P. Dzyubak and E. L. Ritman proposed in a paper in this issue a solution for automation of an adaptive nonsupervised system for tracking tubular objects based on a multiscale framework and a Hessian-based object shape detector. 

We hope that researchers in applied mathematics and medical imaging will find this special issue interesting and useful.

*Yangbo Ye* *Robert J. Plemmons* *James G. Nagy* *Qinian Jin*

